# Approaches for inactivating highly pathogenic avian influenza H5N1 cattle isolate for safe containment level 2 laboratory practices

**DOI:** 10.1128/aem.02356-24

**Published:** 2025-04-10

**Authors:** Lauren Aubrey, Ulises Barron-Castillo, Nathalie Berube, Natalia Pessoa, Leslie Macas Jacome, Jill Trann, Andrew Gentes, Jill Van Kessel, Bryce Warner, Antonio Facciuolo, Yan Zhou

**Affiliations:** 1 Vaccine and Infectious Disease Organization (VIDO), University of Saskatchewan 7235, Saskatoon, Saskatchewan, Canada; 2 Vaccinology and Immunotherapeutics Program, School of Public Health, University of Saskatchewan 7235, Saskatoon, Saskatchewan, Canada; 3 Department of Veterinary Microbiology, Western College of Veterinary Medicine, University of Saskatchewan 7235, Saskatoon, Saskatchewan, Canada; 4 Biochemistry, Microbiology & Immunology, College of Medicine, University of Saskatchewan 7235, Saskatoon, Saskatchewan, Canada; University of Nebraska-Lincoln, Lincoln, Nebraska, USA

**Keywords:** highly pathogenic avian influenza virus, dairy cow H5N1 virus, inactivation, containment level 3, containment level 2

## Abstract

In March 2024, highly pathogenic avian influenza (HPAI) H5N1 was detected in Texas dairy cattle and has since spread to over 500 herds in the United States. Historically, H5N1 transmission to humans has occurred because of contact with infected birds, and over 800 cases have been reported since 2003, with a mortality rate of 52%. Sustained human-to-human transmission of H5N1 has not been observed. HPAI requires physical containment and operational practices to be completed in a containment level 3 (CL-3) laboratory. To safely bring samples containing inactivated HPAI to CL-2 laboratories for further analysis, we tested methods of inactivation for downstream RNA extraction or antibody response assays. Samples containing A/dairy cattle/Texas/24-008749-002/2024 (H5N1 cattle virus) destined for RNA extraction were incubated with Buffer AVL (Qiagen) with 95% ethanol, or Buffer RLT (Qiagen) with 70% ethanol. Samples tested for antibody assays, serum, and milk containing HPAI were incubated with 0.5% vol/vol Triton X-100 at 60°C. We found that Buffer AVL with 95% ethanol inactivated H5N1 cattle virus in supernatant from infected cells, milk, blood, and urine. Buffer RLT with 70% ethanol inactivated H5N1 cattle virus in infected cell pellet, spiked milk, blood, urine, and tissue. Finally, incubation with 0.5% vol/vol Triton X-100 followed by a 30 minute heat treatment at 60°C completely inactivated the H5N1 cattle virus in whey and serum. This work is essential for allowing the safe transfer of H5N1 samples produced in CL-3 to lower containment laboratories for downstream analyses.

**IMPORTANCE:**

Historically, human infections from highly pathogenic avian influenza (HPAI) have occurred from contact with infected birds, with estimated mortality rates of 52%. Recently, this virus has spilled over into many mammalian species and has rapidly spread between dairy cattle herds in the United States, causing multiple human infections after exposure to infected cows. Characterization of this virus is imperative for reducing risk to humans. Work with live HPAI virus must be undertaken in containment level 3 facilities, which limits the amount and type of work that can be done due to time-consuming biosafety procedures and lack of equipment. In this article, we outline how to effectively inactivate HPAI to enable safe work in containment level 2 facilities and facilitate more efficient work on this pathogen.

## INTRODUCTION

The highly pathogenic avian influenza (HPAI) virus of Eurasian lineage goose/Guangdong was first detected in China in 1996. Viruses of this lineage have rapidly evolved since then, reassorting with other influenza viruses and acquiring new gene mutations that enabled them to become panzootic. This lineage has been classified into eight clades (2.3.4.4a–2.3.4.4h), based on these new genetics (1). Clade 2.3.4.4b has spread globally and was introduced into North America in 2021/2022 during wild bird migration, causing high morbidity and mortality in poultry and wild birds (1–3).

HPAI H5N1 has recently spilled over into many mammalian species including cats, alpacas, seals, and free-living mesocarnivores (4, 5). In March 2024, HPAI H5N1 was detected in Texas dairy cattle, subsequently spreading to over 500 herds in the United States (6). The infected cows showed flu-like symptoms, including fever, reduced feed intake, thick, discolored milk associated with decreased milk production and high levels of virus in milk and mammary tissue (1). This unprecedented spillover event in ruminants underscores the capacity of HPAI H5N1 virus to further expand into a new host, as avian influenza virus infection has never been reported in cattle.

In humans, transmission of HPAI is typically from close contact with infected birds or dairy cattle, or contaminated environments such as farms or live animal markets (7). Sustained human-to-human transmission of H5N1 has never been observed (8). As of 19 November 2024, the CDC confirmed 52 cases of human infection with the H5 virus in the United States in 2024, 31 of which were from exposure to infected dairy cattle, 20 from exposure to poultry, and 1 case in Missouri with no known source of exposure (9). All were mild cases with symptoms including conjunctivitis and typical flu symptoms such as fever, chills, coughing, sore throat, and runny nose (9–12). In November 2024, the first locally acquired human case of H5N1 in Canada was reported (13). Previous human case data gave case fatality rates of around 52% for H5N1; however, that may be an overestimate due to under-reporting of mild cases (8). It is not yet clear whether the dairy cattle HPAI viruses are less pathogenic in humans, or if infections are less severe due to route and dose of infection (14). HPAI is classified as a risk-group 3 pathogen due to its high individual risk and low community risk, so work with HPAI is undertaken in biosafety containment level 3 (CL-3) laboratories (15, 16).

Work in CL-3 laboratories is laborious for workers due to additional personal protective equipment (PPE) and time-consuming decontamination procedures, which limits the amount and type of work that can be done in the laboratory. Since CL-3 laboratories are highly specialized and set up for a specific pathogen or set of pathogens, and equipment cannot be shared between labs, it is not feasible to bring all types of equipment into the CL-3 laboratory. If samples in the CL-3 laboratory can be inactivated, it is therefore beneficial to transport samples to a CL-2 laboratory for further analyses.

Evaluation of viral RNA level by RT-qPCR is a sensitive and practical method for virus detection. For disease diagnosis, understanding HPAI tissue tropism and transmission route in cattle, and studying host response, RNA in milk, nasal discharge, blood, urine, and various tissues often need to be extracted for analysis of viral RNA level and host transcriptomics. Similarly, to understand the systemic and local immunity against HPAI H5N1, the antibody titer in milk and serum needs to be evaluated. In this report, we evaluated the ability of Buffer AVL and RLT, the two commonly used buffers for RNA extraction, for inactivation of HPAI in various body fluids and bovine tissue. We also evaluated Triton X-100 and heat-inactivation ability to inactivate HPAI in milk and serum.

## MATERIALS AND METHODS

### Viruses and cells

Madin-Darby canine kidney (MDCK; ATCC #CRL-2936) cells were used for virus stock propagation and for viral recovery assays after sample inactivation. The MDCK cells were maintained in minimal essential medium (MEM) (Sigma-Aldrich, M4655, St. Louis, MO, USA) containing 10% fetal bovine serum (FBS) (Thermo Fisher Scientific, 16000-044, Ottawa, ON, Canada), and were kept in a humidified 5% CO_2_ incubator at 37°C. H5N1 virus isolate A/dairy cattle/Texas/24-008749-002/2024 (referred to as H5N1 cattle virus hereafter) was obtained from the United States Department of Agriculture and was propagated in MDCK cells in viral growth media (MEM containing 0.2% bovine serum albumin [BSA] [Sigma-Aldrich, A7030, St. Louis, MO, USA] with 1 µg/ml L-[(toluene-4-sulphonamido)-2-phenyl] ethyl chloromethyl ketone [TPCK]-trypsin). All infectious experiments were conducted in biosafety CL-3 at the Vaccine and Infectious Disease Organization at the University of Saskatchewan, Canada, under the guidelines of the Public Health Agency of Canada and the Canadian Food Inspection Agency.

### Preparation of virus-containing samples

To prepare the test articles of viral supernatant and infected cells, MDCK cells (in six-well plate) were infected at an MOI of 1 with H5N1 cattle virus, or mock-infected with MEM as a negative control. Complete cytopathic effect (CPE) was seen at 24 h post-infection (hpi) in the virus-infected well, so the supernatant and cells were harvested from both samples.

To prepare the test articles of tissue sample in Buffer RLT, 30 mg of bovine tissue in 0.3 mL MEM was spiked to a final concentration of 10^6^ 50% tissue culture infectious dose (TCID_50_)/mL of H5N1 cattle virus and homogenized at 4 m/s for 5 min with 2 mm steel beads using the Bead Ruptor ELITE (OMNI International, Kennesaw, GA, USA).

**Fig 1:**
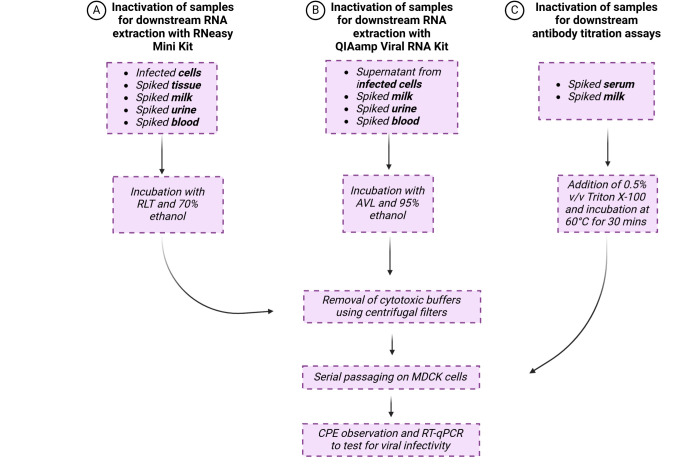
Methods used for testing H5N1 inactivation. H5N1-infected cells and H5N1-spiked test articles (tissue, milk, urine, and blood) were incubated with Buffer RLT for 10 min, and then 70% ethanol was added and incubated for 10 min. Samples were subjected to a 100 kDa Amicon Ultra-4 Centrifugal Filter Unit to remove cytotoxic buffers, then passaged four times on MDCK cells. CPE was observed after each round of amplification, and samples of supernatant after each round were tested for viral RNA using RT-qPCR (**A**). Supernatant from H5N1-infected cells, spiked milk, urine, and blood were incubated with Buffer AVL for 10 min, then 95% ethanol was added and incubated for 10 min. Samples were subjected to a 100 kDa Amicon Ultra-4 Centrifugal Filter Unit to remove cytotoxic buffers, then passaged four times on MDCK cells. CPE was observed after each round of amplification, and samples of supernatant after each round were tested for viral RNA using RT-qPCR (**B**). Blood was collected in serum separator tubes and centrifuged, then the top serum portion was retained and spiked with H5N1. Milk was spiked with H5N1 and then treated with rennet to obtain whey. Spiked serum and whey were then incubated with 0.5% vol/vol Triton X-100 at 60°C for 30 min, then passaged four times on MDCK cells. CPE was observed after each round of amplification, and samples of supernatant after each round were tested for viral RNA using RT-qPCR (**C**). Image created in BioRender.com.

To prepare the test articles of various body fluids, 1 mL of milk, urine, blood, and serum was spiked with H5N1 cattle virus to a final concentration of 10^6^ TCID_50_/mL. Samples were then prepared for either RNA extraction or antibody titration assays (Fig. 1).

### Test article preparation in RLT and AVL

The RNeasy Mini Kit (Qiagen, 74106, Toronto, ON, Canada) was tested with infected cells, spiked tissue, spiked milk, spiked urine, and spiked blood. This kit uses Buffer RLT as a lysis buffer, to which β-mercaptoethanol (β-ME) (Sigma-Aldrich, M6250, St. Louis, MO, USA) was added at 1 µL per 1 mL RLT buffer (17). To the infected cells, 350 µL Buffer RLT was added and incubated at room temperature (RT) for 10 min, then 350 µL 70% ethanol was added and mixed well. To the homogenized tissue or 140 µL of spiked milk, urine, and blood, 600 µL Buffer RLT was added and incubated at RT for 10 min, then 600 µL 70% ethanol was added and mixed well.

The QiaAMP Viral RNA Mini Extraction Kit (Qiagen, 52906, Toronto, ON, Canada) was tested with supernatant harvested from cells infected with H5N1 cattle virus, spiked milk, spiked urine, and spiked blood. They were prepared by following the first two steps provided in the QiaAMP Viral RNA isolation protocol (18). Specifically, spiked milk, urine, and blood were diluted 1:4 in calcium- and magnesium-free phosphate-buffered saline (PBS), then 140 µL of sample was mixed with 560 µL Buffer AVL and incubated at RT for 10 min (Step 1). The supernatant from infected cells was not diluted. Next, 560 µL of 95% ethanol was added and mixed by pipetting, and incubated at RT for 10 min (Step 2).

### Removal of cytotoxic buffers using centrifugal filters

**Fig 2:**
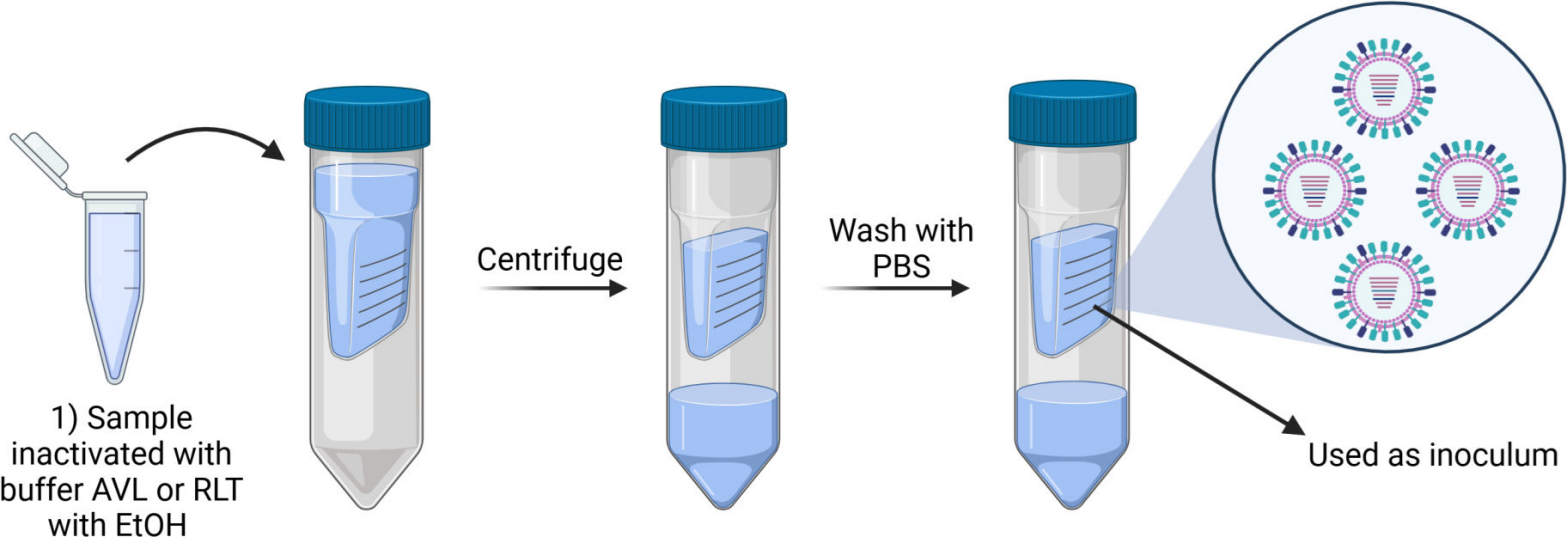
Process of filtration through centrifugal filters. Samples in Buffer RLT and 70% ethanol or Buffer AVL and 95% ethanol were adjusted to 4 mL with PBS and applied to an Amicon Ultra-4 Centrifugal Filter Unit (100 kDa, 4 mL). The Amicon tubes were centrifuged at 4,000 rpm for 5 min, and the flow through was discarded. 4 mL PBS was added to the sample reservoir, and the tube was centrifuged again. This was repeated until 100–200 μL remained in the filter concentrate (top component of the Amicon tube), and this fraction was then used as inoculum for testing of residual infectivity of H5N1 particles. Image created in BioRender.com.

Samples in Buffer AVL/ethanol or RLT/ethanol were adjusted to 4 mL with PBS and added to the sample reservoir of an Amicon Ultra-4 Centrifugal Filter Unit (100 kDa, 4 mL) (Sigma-Aldrich, UFC810008, St. Louis, MO, USA). The Amicon tubes were centrifuged at 4,000 rpm for 5 min and the flow through was discarded. An additional 4 mL of PBS was added to the sample reservoir and centrifuged again. This was repeated until 100–200 μL remained in the filter unit (top portion) of the Amicon tube (Fig. 2).

### Sample preparation for antibody titration

Rennet from *Mucor miehei* (0.125 µg/mL) (Sigma Aldrich, R5876, St. Louis, MO, USA) was added to spiked milk and incubated at 37°C for 5 min until milk curdled. The milk was centrifuged at 1,300 × *g* for 5 min, which resulted in the formation of three fractions: a thin layer of milk fat at the top, a liquid whey fraction, and a pellet containing casein. The top fraction of fat was carefully removed by pipetting before the whey was collected.

Blood was collected in serum separator tubes and allowed to clot before centrifuging at 2,000 rpm for 10 min. The top serum fraction was retained.

Triton X-100 was added to whey and serum to achieve a final concentration of 0.5% (vol/vol) (Sigma Aldrich, 648463, St. Louis, MO, USA), and both were incubated at 60°C for 30 min.

### Testing for residual infectivity of H5N1 particles

MDCK cells were seeded in 12-well plates at 2 × 10^5^ cells per well and incubated at 37°C 5% CO_2_ overnight. The test articles, positive control, and negative control were added to the cells in triplicate (round 0). Inoculum was given 1 h for absorption at 37°C and then media were replaced with MEM containing 0.2% BSA and 1 µg/mL TPCK-trypsin. The cells were incubated at 37°C with 5% CO_2_ for 3 days. The supernatant was harvested (round 1) and centrifuged to remove any cells in suspension (300 × *g* for 8 min). This supernatant was then used as inoculum in a second round of infection. The process was repeated three times more for four total rounds of infection. Cytopathic effects were observed daily and recorded on day 3 post-infection.

### Measurement of total viral RNA

On day 0 of the first round of amplification and day 3 post-infection of each round, supernatant was collected to determine whether viral RNA could be detected. RNA was extracted from supernatant as per QiaAMP viral RNA kit.

A total of 140 µL of supernatant was transferred to 560 µL of Buffer AVL and incubated at RT for 10 minutes. Then, 560 µL of 95% ethanol was added and mixed by pipetting and incubated at RT for 10 min. 630 µL of the solution was transferred to the QIAamp mini spin column and centrifuged for 15 s at 6,000 × *g* for 1 min. Flow-through was discarded, and the process was repeated until all of the sample had been passed through the column. 500 µL of AW1 buffer was added to the column and centrifuged at 6,000 × *g* for 1 min in a new collection tube, with flow-through discarded. 500 µL of AW2 buffer was added to the column and centrifuged at full speed for 3 min, with flow-through discarded. The column was centrifuged empty once for 1 min at full speed to remove residual wash buffer, then 50 µL of elution buffer was added directly to the center of the column membrane and incubated for 1 min before centrifugation in a clean microcentrifuge tube.

**Fig 3:**
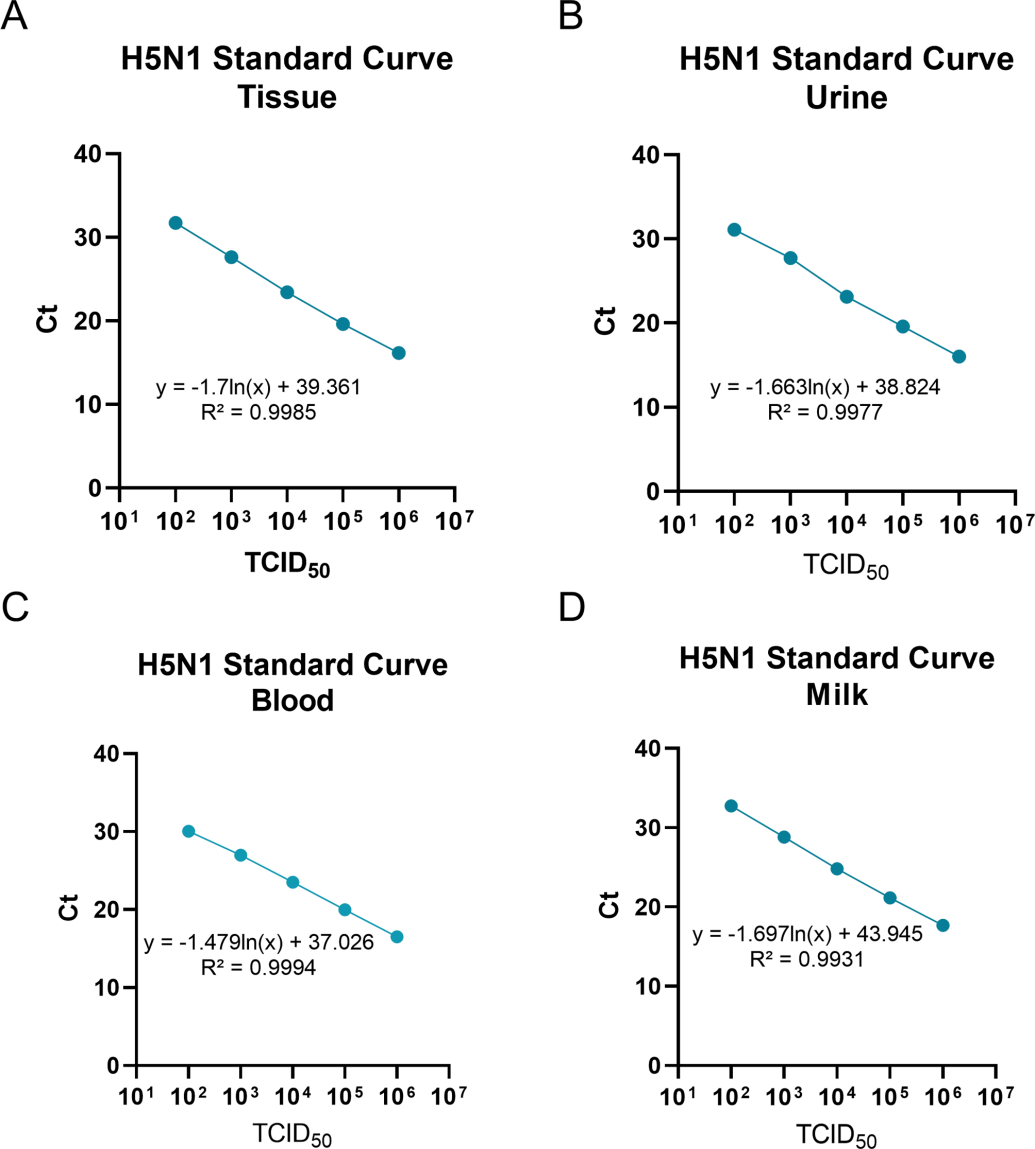
Standard curve for quantification of H5N1 viral load by qPCR in bovine tissue, urine, blood, and milk. RNA from bovine tissue, urine, blood, and milk spiked with 10^6^ TCID_50_ HPAI was extracted using the Qiagen RNeasy Mini Kit (**A**) and the Qiagen QIAamp Viral RNA Kit (**B–D**). RNA was diluted 10-fold using ddH_2_O and RT-qPCR was run using the NEB Luna qPCR Kit and primer-probe sets specific for the influenza A virus M gene. The mean of the Ct values obtained from the duplication of each dilution was used to make a standard curve for each plate.

Once extracted, 5 µL of the sample was subjected to qPCR with Luna qPCR Kit (NEB, E3006L, Whitby, ON, Canada) on the StepOne Plus Real-Time PCR System (QuantStudio6 Applied Biosciences) using a primer-probe set specific for influenza A virus M gene. Forward primer: GGCCCCCTCAAAGCCGA, Backward primer: CGTCTACGYTGCAGTCC, Probe: 5 (FAM)-TCACTGGGCACGGTGAGCGT-3′ (MGBNFQ) (IDT, Coralville, IA, USA). A standard curve was created with RNA extracted from samples spiked with known tittered H5N1 virus for each sample type: MEM, tissue, urine, blood, and milk as a positive control. The resulting Ct values were recorded (Fig. 3).

### Neutralization assay with inactivated milk

To verify the antibody activity in milk after heat treatment, milk was collected from a cow prior to and 28 days after intra-mammary challenge with the H5N1 cattle virus. Four replicate samples of milk were inactivated at either 56°C or 60°C to compare virus neutralizing antibody titers after heat treatment at the two different temperatures. MDCK cells (3.5 × 10^4^) were plated into 96-well plates. Milk was treated with rennet and either 56°C or 60°C heat treatment. Twofold dilutions of milk were added to a 96-well plate in quadruplicate, and 60 µL of these dilutions was incubated with 60 µL of H5N1 cattle virus diluted to 2 × 10^3^ TCID_50_/mL at 37°C for 1 h. A total of 100 µL of this mixture was then added to the MDCK cells for 1 h of adsorption at 37°C, and then replaced with viral growth medium. CPE was documented at 72 hpi, and the neutralizing antibody titer was taken as the highest dilution of each milk sample that completely protected the cells from CPE in at least two out of four wells.

### Biosafety statement

Work with HPAI H5N1 was conducted in Vaccine and Infectious Disease Organization (VIDO) CL3 and CL3–Large Animal Facility, in compliance with the Canadian Biosafety Standard, Third Edition, under the authority of the Public Health Agency of Canada and the Canadian Food Inspection Agency.

## RESULTS

### Viral inactivation with Buffer RLT + 70% EtOH

**Fig 4:**
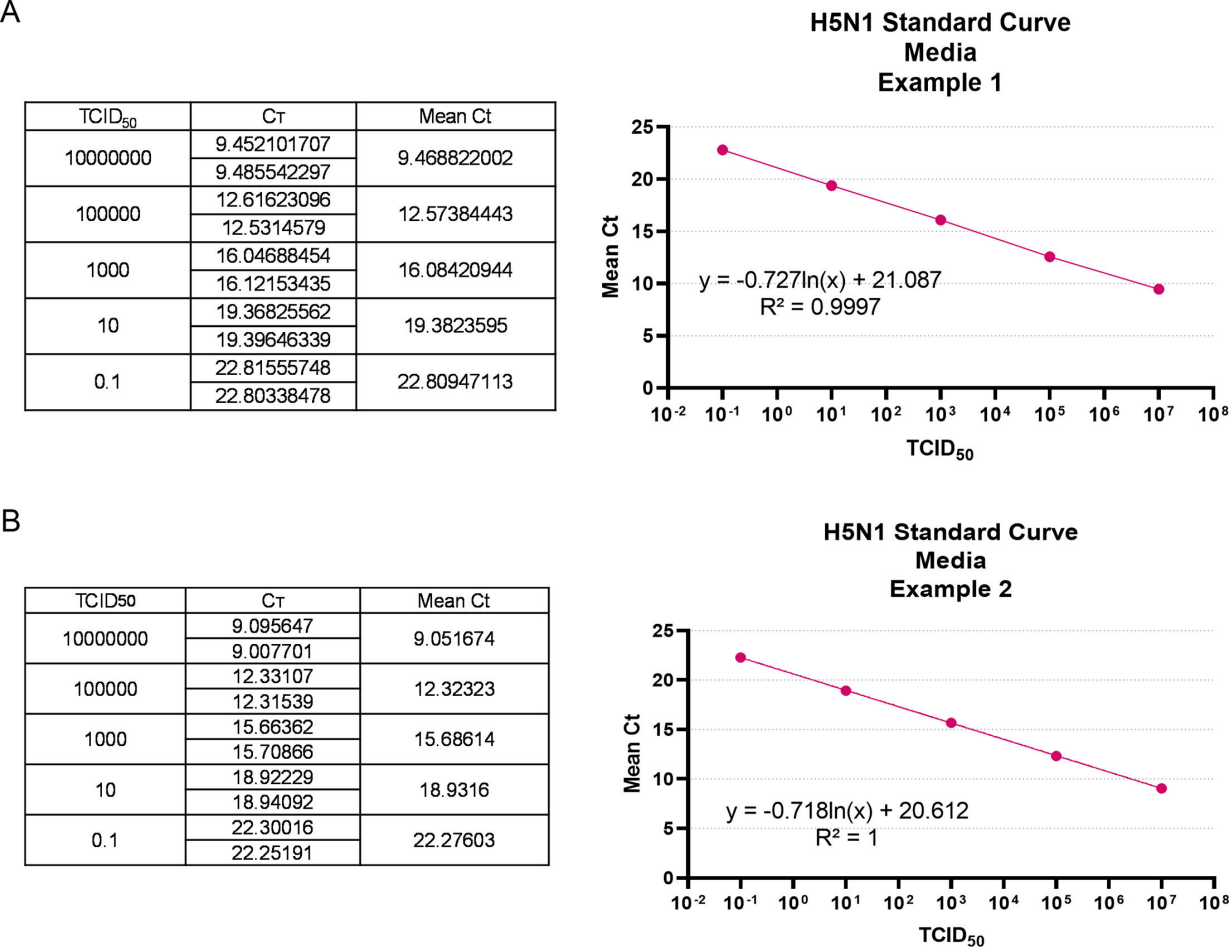
Standard curve for quantification of H5N1 viral load by qPCR. RNA from 10^7^ TCID_50_ of H5N1 cattle virus in 140 µL MEM was extracted using the QIAamp Viral RNA Kit and diluted 100-fold in sterile water to generate a standard curve. This RNA was loaded in duplicate on two separate 96-well plates (A and B, respectively) along with the master mix for RT-qPCR using the NEB Luna qPCR Kit and primer-probe sets specific for the influenza A virus M gene. The mean of the Ct values obtained from each dilution was used to make a standard curve for each plate.

**Fig 5:**
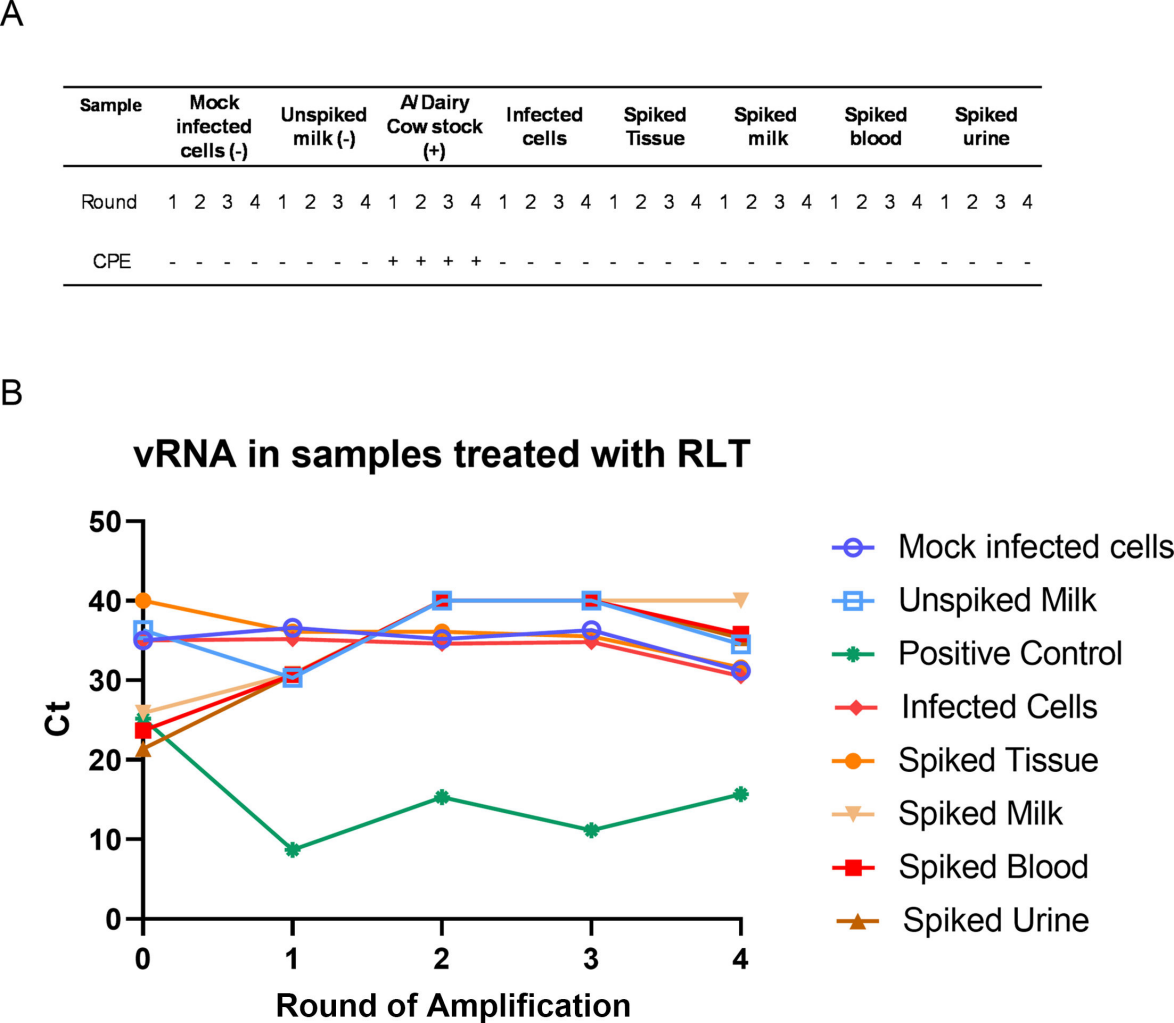
CPE and vRNA in Buffer RLT-inactivated samples. Treated samples were used as inoculum on MDCK cells and adsorbed for 1 h before being replaced with viral growth media. For positive control, cattle H5N1 virus was used to infect MDCK cells. Three days post-infection, supernatant was harvested and used as inoculum on a fresh set of MDCK cells. This was repeated three times for four total rounds of amplification. Observation of CPE in MDCK cells 3 days post-infection in each round (**A**). Supernatant was harvested on day 3 post-infection during each round of infection and tested for vRNA using probes specific for influenza M protein in RT-qPCR (**B**).

The standard curve on each plate varies slightly (Fig. 4). Multiple rounds of experiments showed 10^7^ TCID_50_ of HPAI extracted from media yield a Ct value of 9.0–9.5; 0.1 TCID_50_ of virus yields a Ct value of 22.3–22.8. RNA extracted from negative control supernatant resulted in a Ct value ranging between 31.2 and 36.3. Thus, in our system, a Ct value greater than 30 is deemed negative for the presence of infectious virus, as RNA after each round of infection was extracted from supernatant. For all samples treated with Buffer RLT and 70% ethanol, no CPE was observed in any round of amplification (Fig. 5A). No decrease in Ct value was seen in round 1 compared to round 0 (within the range of Ct 1–30), and Ct values were greater than 30 (Fig. 5B). The CT value of the positive control sample decreased significantly in round 1 compared to round 0 and elevated in rounds 2–4 of amplification. We rationalize this is due to the generation of defective interfering (DI) particles in later rounds of amplification.

### Viral inactivation with Buffer AVL + 95% EtOH

**Fig 6:**
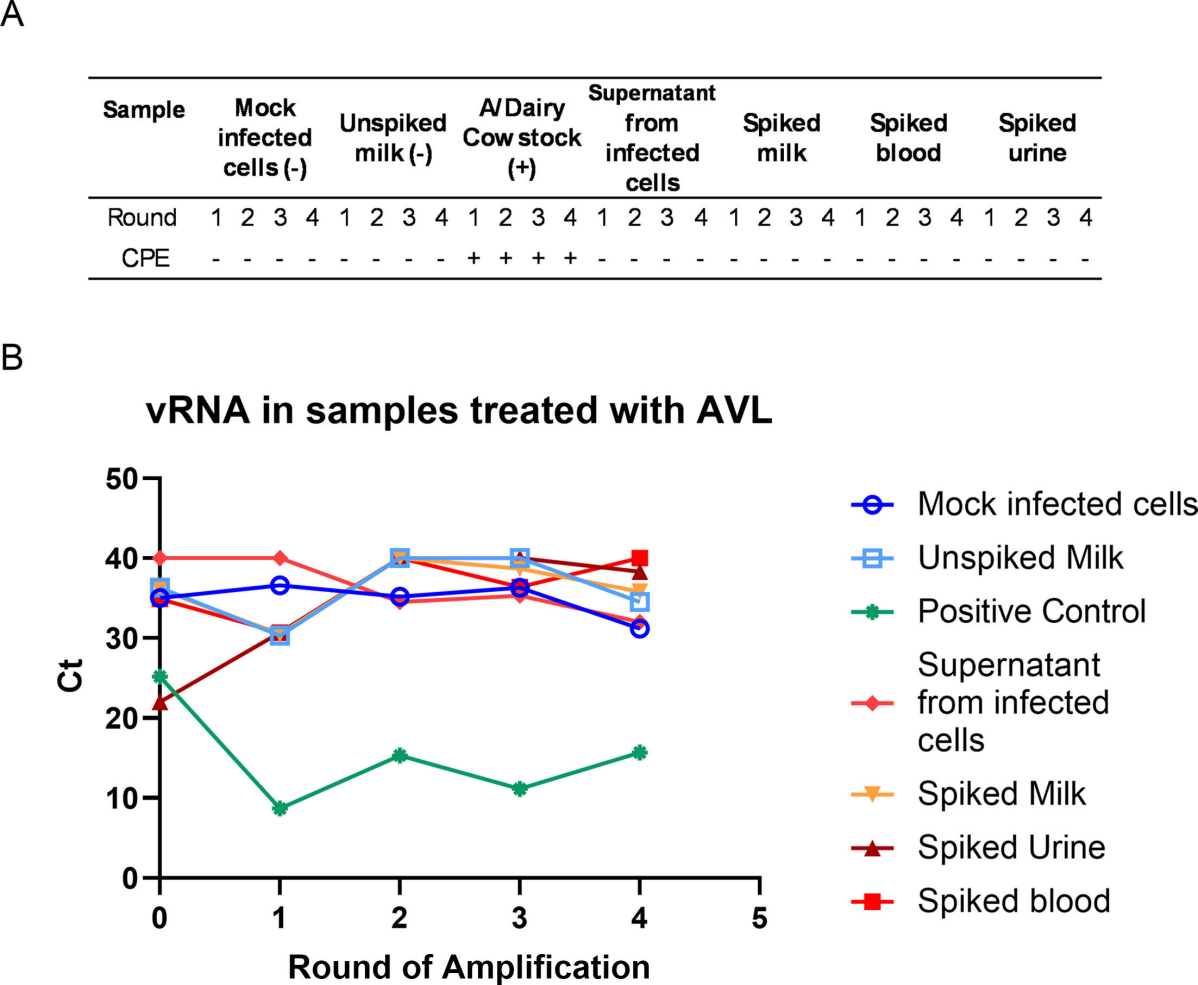
CPE and viral RNA in Buffer AVL-inactivated samples. Treated samples or cattle H5N1 virus (positive control) were used as inoculum on MDCK cells and adsorbed for 1 h before being replaced with viral growth media. Three days post-infection, supernatant was harvested and used as inoculum on a fresh set of MDCK cells. This was repeated three times for four total rounds of infection. Observation of CPE in MDCK cells 3 days post-infection in each round (**A**). Supernatant was harvested on day 3 post-infection during each round of infection and tested for vRNA using probes specific for influenza M protein in RT-qPCR (**B**).

For all samples treated with Buffer AVL and 95% ethanol, no CPE was observed in any round of amplification (Fig. 6A). No decrease in Ct value was seen in round 1 compared to round 0 (within the range of Ct 1–30), and Ct values were greater than 30 (Fig. 6B).

### Viral inactivation with heat treatment and Triton X-100

**Fig 7:**
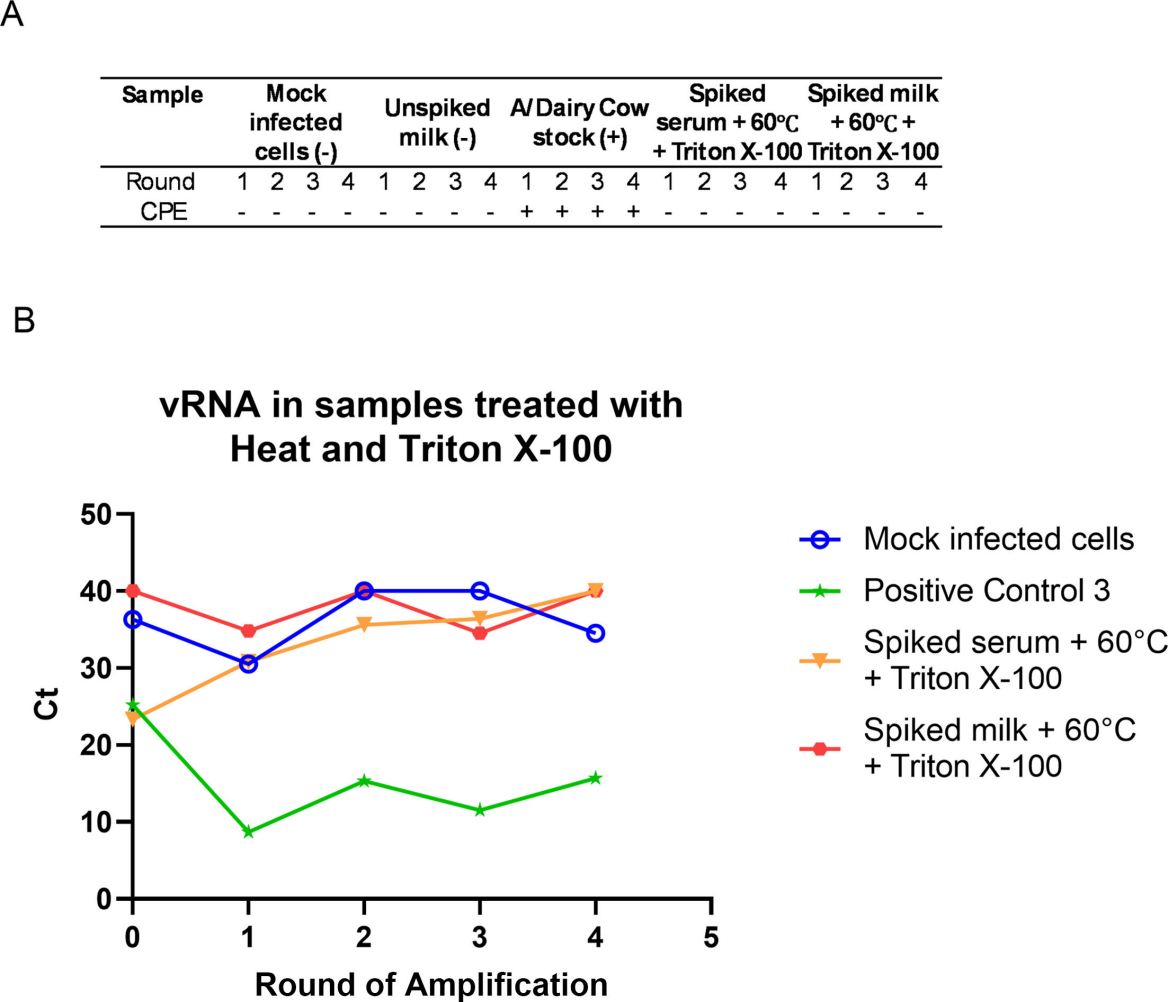
CPE and Viral RNA in heat and Triton X-100 inactivated samples. Treated samples or cattle H5N1 virus (positive control) were used as inoculum on MDCK cells and adsorbed for 1 h before being replaced with viral growth media. Three days post-infection, supernatant was harvested and used as inoculum on a fresh set of MDCK cells. This was repeated three times for four total rounds of infection. Observation of CPE in MDCK cells 3 days post-infection in each round (**A**). Supernatant was harvested on day 3 post-infection during each round of infection and tested for vRNA using probes specific for influenza M1 gene in RT-qPCR (**B**).

For all samples treated at 60°C for 30 min with 0.5% vol/vol Triton X-100, no CPE was observed in any round of amplification (Fig. 7A). For both serum and whey, there was no decrease in Ct value between round 1 compared to round 0 (within the range of Ct 1–30), and Ct values were greater than 30 (Fig. 7B).

### Virus neutralizing antibody assay using heat-inactivated milk

To examine whether 60°C heat treatment interfered with antibody binding, a viral neutralization assay was conducted. In day 0 milk samples, neutralizing antibody titers were 0. In milk collected 28 days post-challenge, titers ranged from 40 to 80 (Table 1). From the samples inactivated at 56°C and 60°C, two samples showed the same neutralization titer after heat treatment with both temperatures. One went up by one dilution factor from 20 to 40, and one went down by one dilution factor from 80 to 40 when inactivating at 60°C as compared to 56°C (Table 2).

**TABLE 1:** Virus neutralizing antibody titers in milk from infected cows^*a*^

Day post-challenge	Sample number	Neutralizing antibody titer in milk
0	Sample 1	0
	Sample 2	0
	Sample 3	0
	Sample 4	0
28	Sample 5	80
	Sample 6	40
	Sample 7	80
	Sample 8	80

^*a*^ Milk was collected from cows before infection and 28 days after infection with H5N1. Milk was treated with rennet; resulting whey was incubated at 60°C for 30 min and then used in a neutralization assay.

**TABLE 2:** Comparison of virus neutralizing antibody titer in milk inactivated at 56°C and 60°C^*a*^

Sample	56°C	60°C
A	80	80
B	80	40
C	20	20
D	20	40

^*a*^ Milk was collected from infected cows and treated with rennet. The resulting whey was incubated at either 56°C or 60°C for 30 min and then used in a neutralization assay.

## DISCUSSION

In this study, we wanted to test methods for inactivating HPAI in samples while preserving the integrity of the biological sample for downstream analyses.

Buffer AVL and RLT contain high concentrations of guanidine thiocyanate, which is a chaotropic salt that denatures macromolecules and provides stability for RNA during the extraction process (19, 20). Avelin et al. showed that Buffer RLT completely inactivated HPAI in infected cell pellet (21). A study with the Ebola virus showed that the addition of Buffer AVL alone did not completely inactivate the virus, but when combined with ethanol, the virus was completely inactivated (22). As the addition of ethanol to the sample is part of the protocol in both Qiagen RNA extraction kits, we tested the inactivation of the virus after the addition of both Buffer AVL with 95% ethanol and Buffer RLT with 70% ethanol as outlined in the kit protocols. The RNeasy kits are recommended for isolating RNA from cells and tissues, so cells infected with HPAI as well as tissue were used as test samples with Buffer RLT and 70% ethanol. We also tested milk, blood, and urine spiked with HPAI with Buffer RLT and 70% ethanol in case Buffer AVL and 95% ethanol could not completely inactivate the virus, since there are cells, protein, and lipids in this body fluid. The QIAamp Viral RNA Mini Kit is recommended for isolating RNA from cell-free body fluids, so supernatant from HPAI-infected cells, spiked milk, spiked urine, and spiked blood were used as test samples.

A lower Ct value in RT-qPCR corresponds to a higher amount of viral RNA; therefore, a decrease in Ct value within the detection range on day 3 compared to day 0 would show amplification of viral RNA, indicating the article could be infectious. Some samples such as supernatant from infected cells, spiked milk, and spiked blood treated with Buffer AVL and 95% ethanol, and infected cells and spiked milk treated with Buffer RLT and 70% ethanol started at a higher Ct value than the positive control. We postulate that after complete lysis of the viral particles and disruption of the proteins associated with the viral RNA (vRNA), the vRNA can flow through the filter membrane (MWCO 100 kDa) during centrifugation into the collection tube, which is the fraction that is discarded. However, if the virus particle remains intact due to incomplete lysis or incomplete dissociation from nucleoprotein, it can remain in the filter device concentrate, which is the top fraction of the tube that is used for the inoculum and RT-qPCR. If the viral membrane is disrupted without complete lysis, we postulate that the inactivated virus would also be retained by the filter membrane, which may explain why some samples such as milk, urine, and blood treated with Buffer RLT started round 0 with a Ct similar to the positive control, but show no replication upon passaging on MDCK cells. Standard curves performed with non-inactivated tissue, urine, blood, and milk show that our RT-qPCR can efficiently detect H5N1 in these samples, ruling out a technical issue. All samples treated with either Buffer RLT or Buffer AVL with ethanol increased in Ct values or remained above a Ct of 30 after all four passages on MDCK cells, and no CPE was seen in MDCK cells after any round of amplification. Buffer AVL with 95% ethanol from the QIAamp Viral RNA Mini Kit led to the complete inactivation of HPAI in supernatant from infected cells, milk, urine, and blood. Buffer RLT with 70% ethanol from the RNeasy Mini Kit inactivated HPAI in tissue, milk, blood, urine, and infected cells. As the inactivation was complete, we showed that after inactivation, these sample types can be brought to CL-2 for the latter half of the RNA extraction and downstream analyses in our laboratory.

For inactivation of samples for antibody assays such as ELISA or neutralization assays, incubation at 60°C for 30 min with the addition of 0.5% vol/vol Triton X-100 was tested. The food industry pasteurizes milk at a temperature of 63°C for 30 min, and studies have shown that this completely inactivates milk spiked with a high titer of HPAI H5N1 virus (23). For neutralization assays, it is typical to heat inactivate samples at 56°C for 30 min (24). For our inactivation tests, we chose a temperature at a midpoint of those two temperatures, 60°C for 30 min, to test whether that temperature could inactivate the virus while leaving immunoglobulins functional. Triton X-100 is a surfactant that has been shown to inactivate a wide range of enveloped viruses by disrupting lipid structures, so we tested it in combination with heat treatment (25). As serum and milk are easily accessible biological samples in cows to extract antibodies for immune assays, serum and milk spiked with HPAI were used as test subjects for heat treatment with or without 0.5% v/v Triton X-100. Observation of CPE during serial passaging of these inactivated samples, as well as Ct values from RT-qPCR, indicated that inactivation was complete in serum and milk treated with 0.5% vol/vol Triton X-100 and incubation at 60°C for 30 min. Interestingly, the Ct value for whey as compared to serum and the positive control of serial passaging of stock virus was quite a lot higher at passage 0. RNA extracted from whole milk spiked with virus showed that our RT-qPCR system efficiently detects virus in milk (Fig. 3), so we hypothesize that during the process of generating whey, the virus was lost to either the pelleted curds or trapped in the milk fat layer that was removed. In milk, fat exists as droplets coated in milk fat globule membranes (MFGMs). Triglycerides are produced in the endoplasmic reticulum of epithelial cells in the mammary gland, where they are coated in one layer of phospholipids along with proteins, sphingolipids, and cholesterol (26). When the lipid droplets are released from the apical side of the lactating mammary epithelial cells, they are encased in the phospholipid bilayer of the epithelial cells, along with carbohydrates, glycoproteins, and proteins present in the cell membrane, leading to a triple-layered globule (27). These isolated glycoproteins on MFGMs have been shown to inhibit hemagglutination of *Helicobacter pylori in vitro*, and human MFGM-associated lactadherin inhibited rotavirus binding and infection *in vitro*, likely by acting as a decoy for binding, as removal of sialic acid resulted in the loss of inhibitory activity (28–30). Interestingly, bovine lactadherin did not have the same effect, potentially due to differences in attached oligosaccharides (29). The presence of these glycoproteins on MFGMs may indicate that the influenza virus in our rennet-treated milk may be associated with the lipid fraction where the milk fat globules are located, due to binding to sialic acid receptors on the membranes (31). We also showed that the difference in heat treatment at 60°C compared to 56°C does not have a significant effect on antibody function in neutralization assays.

In conclusion, we showed that lysis Buffers AVL and RLT with 95% ethanol and 70% ethanol, respectively, inactivated HPAI in infected cells, supernatant, tissue, and body fluids while leaving samples intact for RNA extraction and downstream experiments such as RT-qPCR or sequencing. Heat treatment in combination with the addition of 0.5% vol/vol Triton X-100 inactivated HPAI in serum and whey samples without affecting antibody integrity and facilitating downstream antibody assays. This analysis provided information to the laboratory intending to transfer samples from CL-3 to CL-2 for downstream analysis.

## Data Availability

All data supporting the findings of this study are reported in the paper and are available upon request.
